# PM_2.5_ Exposure and Health Risk Assessment Using Remote Sensing Data and GIS

**DOI:** 10.3390/ijerph19106154

**Published:** 2022-05-18

**Authors:** Dan Xu, Wenpeng Lin, Jun Gao, Yue Jiang, Lubing Li, Fei Gao

**Affiliations:** 1 School of Environmental and Geographical Sciences, Shanghai Normal University, Shanghai 200234, China; 1000497916@smail.shnu.edu.cn (D.X.); 1000496184@smail.shnu.edu.cn (Y.J.); 1000459357@smail.shnu.edu.cn (L.L.); 1000496756@smail.shnu.edu.cn (F.G.); 2 Yangtze River Delta Urban Wetland Ecosystem National Field Observation and Research Station, Shanghai 200234, China

**Keywords:** air pollution, PM_2.5_ exposure, health risk, geographic information systems, remote sensing

## Abstract

Assessing personal exposure risk from PM_2.5_ air pollution poses challenges due to the limited availability of high spatial resolution data for PM_2.5_ and population density. This study introduced a seasonal spatial-temporal method of modeling PM_2.5_ distribution characteristics at a 1-km grid level based on remote sensing data and Geographic Information Systems (GIS). The high-accuracy population density data and the relative exposure risk model were used to assess the relationship between exposure to PM_2.5_ air pollution and public health. The results indicated that the spatial-temporal PM_2.5_ concentration could be simulated by MODIS images and GIS method and could provide high spatial resolution data sources for exposure risk assessment. PM_2.5_ air pollution risks were most serious in spring and winter, and high risks of environmental health hazards were mostly concentrated in densely populated areas in Shanghai-Hangzhou Bay, China. Policies to control the total population and pollution discharge need follow the principle of adaptation to local conditions in high-risk areas. Air quality maintenance and ecological maintenance should be carried out in low-risk areas to reduce exposure risk and improve environmental health.

## 1. Introduction

Air pollution has been considered a global health priority regarding several Sustainable Development Goals (SDGs), such as Goal 3 (Ensure healthy lives and promote well-being for all at all ages) [1,2]. Fine particulate matter (a diameter of less than 2.5 μm, PM_2.5_) pollution is a common type of air pollution in recent years. According to the China Ecological Environmental Bulletin [3,4,5,6,7], 40~75% cities in China exceeded the standard for ambient air quality (PM_2.5_ < 75 µg/m^3^), and on 45~80% of days, PM_2.5_ as the primary pollutant exceeded the standard. One study by Son et al. found that short-term exposure to PM_2.5_ was positively associated with increased risk of mortality [8]. Other studies indicated that the long-term chronic effects of PM_2.5_ may cause cardiovascular diseases such as lung cancer, myocardial infarction, and myocardial ischemia [9,10,11], and it was an important cause of acute triggering of common respiratory system diseases such as asthma, bronchitis, rhinitis, and upper and lower respiratory tract infections [12,13,14]. Long-term exposure to PM_2.5_ pollution may lead to slow growth, slow neurological development, and brain dysfunction in children [15,16]. In addition, it may lead to depression and pessimism, and even suicidal behavior [17,18,19]. Due to its critical impact on health, exposure to PM_2.5_ and health risk assessment has been a critical concern for ensuring healthy lives and promoting well-being.

There are various data and methods for quantifying ambient air pollution in recent years. Remote sensing (RS) and Geographic Information Systems (GIS) methods have been increasingly used in environment and health research, with corresponding improvements in the use and accessibility of multi-temporal satellite-derived environmental data [20,21]. Some studies estimated PM_2.5_ concentration and spatial-temporal distribution from air quality monitoring stations using spatial interpolation techniques from GIS [22,23,24], but this approach ignored the uneven distribution of monitoring stations and the rough spatial resolution of the data. The rapid development of RS technology and advanced satellite sensors (with particulate matter detection instruments) addressed this issue. Many satellites (e.g., GOES, Terra, Aqua, METOP, PARASOL) equipped with multifunctional sensors (e.g., MODIS, AVHRR, SeaWiFS, POLDER) greatly promoted the development of Aerosol Optical Depth (AOD, one of the most important parameters of aerosols) inversion by remote sensing imagery interpretation and processing [25,26,27,28,29]. Kaufman introduced the dark target algorithm of AOD inversion based on band relationships [30], and Levy et al. improved the accuracy of AOD inversion of the algorithm [31]. Tanré and Holben developed the structural function method by using atmospheric transmittance to obtain aerosol information [32,33]. Hsu et al. directly adopted surface reflectance data on visibles band to retrieve AOD using the deep blue algorithm [34,35]. Lyapustin et al. proposed a new correction algorithm for AOD inversion, the multi-angle atmospheric correction algorithm, using time series analysis and image processing technology for atmospheric correction and AOD inversion [36,37].

Many researchers found a specific relationship between AOD and PM_2.5_ that could be estimated by some methods from AOD to PM_2.5_ [38,39,40]. The model correction method used different models to correct various influencing factors and simulate the proportion relationship between AOD and PM_2.5_, such as the atmospheric chemical transport model (GEOS-Chem) [41,42,43] and the atmospheric boundary layer model (RAMS) [44]. However, this method ignored the physical mechanism between AOD and PM_2.5_. Both aerosol type and vertical distribution lead to differences in scattering extinction. The mechanism correction method solved this problem and obtained the stable aerosol extinction coefficient by vertical correction and scattering extinction correction for PM_2.5_ estimation [45,46,47,48]. However, this method was highly dependent on setting physical mechanism parameters, and these parameters are different in different areas. In order to monitor and estimate PM_2.5_ in real time, it is necessary to update these physical mechanism parameters in time. In addition, the statistical model method established linear or nonlinear statistical models between the AOD and PM_2.5_ based on various meteorological or environmental elements (wind speed, direction, position, humidity, height, etc.), such as multiple linear regression model (GLM) [49,50], generalized summation model (GAM) [51,52], and geographically weighted regression model (GWR) [53,54,55,56,57]. Some researchers combined several statistical models to construct multilevel statistical models to estimate PM_2.5_ concentration. In recent years, the machine learning (ML) method has been widely used to associate AOD with PM_2.5_, incorporating big geographic spatial-temporal data as examples and with self-supervision and training functions [58,59]. Although this method had high accuracy in estimating results and could deal with the complex relationship between AOD and PM_2.5_, it required multistep processing of training samples in advance and relevant operations on physical and chemical mechanisms, which increased the difficulty of use to a certain extent [60]. Many methods have associated AOD with PM_2.5_, each with its characteristics.

To explore the relationship between PM_2.5_ air pollution and health exposure risk, one study by Zou et al. found a spatial pattern of population exposure to air pollution by constructing a relative exposure risk assessment model [61]. Tong et al. showed that population density data and the relative exposure risk assessment model could more reasonably represent the relationship between PM_2.5_ pollution and environmental health [62]. Lu et al. developed a personal mobility model to quantify long-term air pollution exposure for individuals [63]. Park developed an air dispersion model based on a large sample of travel-activity diary data to assess personal exposure to PM_2.5_ [64]. These studies confirmed the applicability to and efficiency of remote sensing data and GIS for exposure to PM_2.5_ air pollution and environmental health. However, risk assessment results from these studies were not sufficiently accurate owing to the limited availability of high spatial resolution data for PM_2.5_ and population density.

Toward this end, this study presents a framework that incorporates high spatial resolution remote-sensing images and geographic spatial-temporal data to retrieve AOD and estimate PM_2.5_ based on the GIS platform. The Enhanced Dark Target Algorithm (EDTA) is introduced to retrieve daily AOD from MODIS images, improving the spatial resolution at 1 km grid level. Spatial-temporal seasonal models are used to estimate PM_2.5_ concentration using geospatial-temporal data and GIS spatial analysis methods. Then, we assess the exposure risk to PM_2.5_ pollution using high-accuracy population density data and the relative exposure risk models. Finally, we discuss the usage and deficiency of assessment results and offer our expectations for future application. We look forward to provide scientific reference for improving urban atmospheric pollution and living environment.

## 2. Materials and Methods

### 2.1. Study Area

**Figure 1:**
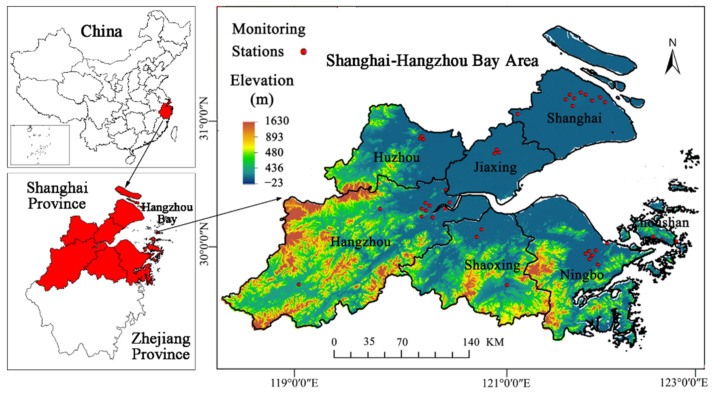
The location and elevation map of the study area, SHB.

SHB is located in eastern China and the north Pacific coastal area (28°51′~31°53′ N, 118°21′~123°25′ E) and is an important part of the integrated development of the Yangtze River Delta, China. From north to south, it contains seven cities, including Shanghai, Jiaxing, Huzhou, Hangzhou, Shaoxing, Ningbo and Zhoushan. Its total land area is about 52,300 km^2^, and its population is about 54.41 million (2018 Statistical Yearbook of China). Belonging to the northern subtropical region, SHB is characterized by a mild and humid climate with abundant rainfall. The terrain is higher in the southwest and lower in the northeast; plains, hills and mountains cover the most part (Figure 1). In 2018, SHB had five of the top 100 cities (in comprehensive strength) in China: Shanghai (no. 2), Hangzhou (no. 5), Ningbo (no. 20), Shaoxing (no. 34), Jiaxing (NO. 39), reflecting the highly developed policy, economy, society, culture, and ecology in SHB.

### 2.2. Data Source

#### 2.2.1. Vector and Elevation Data

The data on the region boundaries and elevation were from the Resource and Environmental Sciences and Data Center, China (ESDC, http://www.resdc.cn/Default.aspx, accessed on 18 May 2019).

#### 2.2.2. Remote Sensing Data

Terra MODIS L1B data (Level 1, with Global Certification Level) were applicable and reliable for China [65,66] at a spatial resolution of 1 km ×1 km. Excluding a period of no data from 19 to 27 February, there were 1 to 3 image data every day, for a total of 827 images of original data from SHB from 1 January to 31 December 2016. Data were downloaded from NASA LAADS Web to retrieve AOD (LAADS, https://ladsweb.nascom.nasa.gov/, accessed on 4 December 2019).

#### 2.2.3. Ground-Based AOD Observation Data

The observation error for ground-based AOD from AERONET is 0.01~0.02, which can be used directly for the correction of estimated AOD [67]. The Version 3 and Level 1.5 data (data after cloud processing) in 2016 were downloaded from the Aerosol Robotic Network (AERONET, http://aeronet.gsfc.nasa.gov/, accessed on 18 May 2019) for verifying the accuracy of retrieved AOD. We finally chose Level-1.5 AOD data from two stations (SONET_Shanghai and SONET_Zhoushan) according to the actual situation in SHB (Table 1).

#### 2.2.4. Ground-Level PM_2.5_ Observation Data

Based on China national standards and specifications for ambient air quality assessment, daily observation data on PM_2.5_ concentrations (unit: µg/m^3^) at air quality monitoring stations were derived from the official website of the China Environmental Monitoring Center for exploring the optimal relationship between retrieved AOD and PM_2.5_ observation data (CEMC, http://106.37.208.233:20035/, accessed on 23 December 2019). There were 41 monitoring stations in SHB, and specific information is in Table 2.

#### 2.2.5. Population Density Data

The population density data came from the open population data set platform Worldpop (https://www.worldpop.org/, accessed on 23 December 2019), with a spatial resolution of 1 km × 1 km. Based on remote sensing images and geospatial-temporal big data, this data set used the random forest algorithm to simulate the spatial-temporal distribution of the population density, comprehensively considering land cover and land use, residential areas, roads, buildings, public facilities, night lights, vegetation, geographic and geomorphic conditions, and so on [68].

### 2.3. Methods

#### 2.3.1. Enhanced Dark Target Algorithm (EDTA)

The dark target algorithm (DTA) is an operational algorithm with high inversion accuracy and maturity. Kaufman found a good linear relationship between surface reflectivity in the near-infrared band (2.1 µm) and the visible red band (0.66 µm) and visible blue band (0.47 µm) [69]. The near-infrared band is affected very little by aerosols, and its apparent reflectivity can be approximated as surface reflectivity [26]. The surface reflectivity of the visible red and blue bands can be estimated with a linear calculation equation:

(1) $$\rho_{r}=\frac{1}{2}*\rho_{n}$$

(2) $$\rho_{b}=\frac{1}{4}*\rho_{n}$$

where $\rho_{r}$ is the surface reflectivity of the visible red band (0.66 μm); $\rho_{b}$ is the surface reflectivity of the visible blue band (0.47 μm); and $\rho_{n}$ is the surface reflectivity of near-infrared band (2.1 μm).

Apparent reflectivity is the result of surface reflectivity and atmospheric reflectivity, as shown in Equation (3). The apparent reflectivity information of the visible red and blue bands can be obtained from satellite remote sensing data, and the surface reflectivity of the visible red and blue bands can be obtained after removing the linear estimation, which gives the atmospheric reflectivity [70]:

(3) $$\rho^{*}\left(\mu ,\varphi ,\mu_{0},\varphi_{0}\right)=\rho_{a}\left(\mu ,\varphi ,\mu_{0},\varphi_{0}\right)+\frac{T\left(\mu_{0}\right)T\left(\mu \right)\rho }{1-\rho s}$$

where $\rho^{*}$ is apparent reflectivity; μ is the cosine of the satellite zenith angle, $\mu_{0}$ is s the cosine of the solar zenith angle, φ is the satellite azimuth angle, $\varphi_{0}$ is the solar azimuth angle; $\rho_{a}$ is atmospheric reflectivity; $T\left(\mu_{0}\right)$ is the total transmittivity from the sun to the earth’s atmosphere; $T\left(\mu \right)$ is the total transmittivity from the earth’s surface to the satellite’s atmosphere; ρ is surface reflectivity; and s is the spherical surface albedo of the atmosphere.

**Figure 2:**
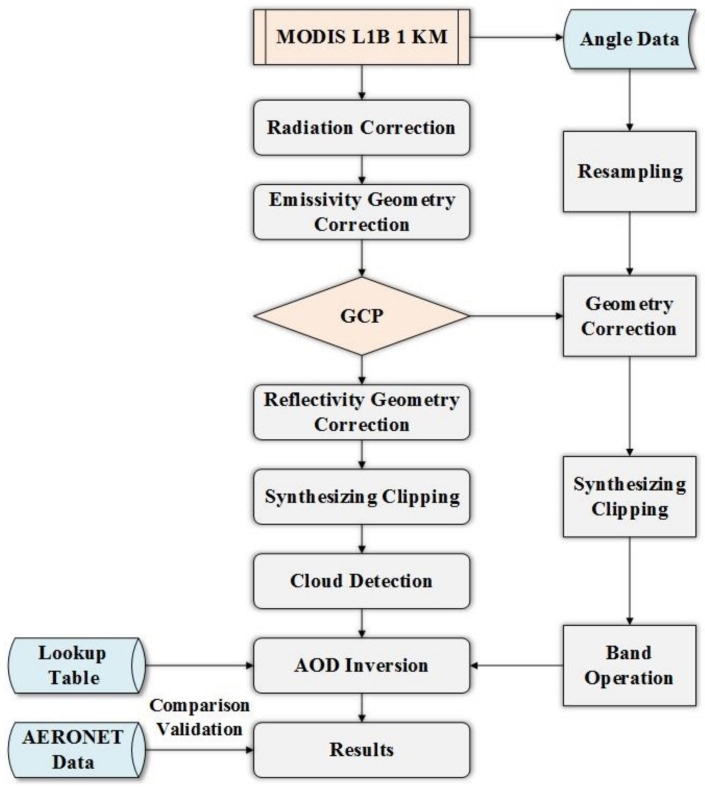
The flow chart of AOD inversion.

Based on the basic dark target algorithm (DTA), we introduced the enhanced dark target algorithm (EDTA) to retrieve the AOD according to the actual situation in SHB. The basic inversion process included radiometric correction, geometric correction, resampling, composition and clipping, cloud detection and elimination, Lookup Table (LUT) setting, and accuracy verification of the inversion results (Figure 2). We improved the inversion algorithm, especially in building LUT.

Geometry correction. MODIS L1B 1 km data (MOD02_1KM) contains emissivity and reflectivity files and angle data (sensor zenith and sensor azimuth of the satellite, solar zenith and solar azimuth of the sun), which are obviously different types. We employed ENVI software to resample the row and column numbers of angle data set from 271 × 406 to 1354 × 2030 (e.g., emissivity and reflectivity files) before correction. The HDF file was used to generate ground control points (GCPs). The correction model was Triangulation, and the resampling method was Bilinear.

Band operation and cloud detection. The Layer Stacking tool was adopted for synthesizing geometry files and angle data after correction. The stacked order of emissivity and reflectivity files (as well as angle data) affected the results. The reflectivity file must be placed in up and the emissivity file in down. In fact, angle data had been expanded 100 times in HDF files, so it should have been multiplied by 0.01 during band operation. In order to remove the influence of cloud reflection, absorption and scattering noise, the Cloud Detection tool was installed.

LUT Setting and AOD retrieval. The accuracy of LUT determines the accuracy of AOD retrieval to a certain extent. A 6S Radiation Transmission model was used to distinguish various surface types and observation bands. Different parameters of atmospheric aerosol and observation parameters were preset for radiation transmission calculation to obtain inversion results. After geometric correction, band operation, band clipping, cloud detection, and invalid value elimination, the results for emissivity, reflectance, and angle data set were combined with the LUT to perform aerosol inversion calculation, and we obtained AOD inversion values. According to geographical features and the actual situation in SHB, we improved the related model and enhanced the LUT setting in the geometric parameters (Table 3) to produce the enhanced dark target algorithm (EDTA).

Validation. The ground-based AOD from AERONET data was used to verify the accuracy of the retrieved AOD. We set the value of AOD at 550 nm wavelength (0, 0.25, 0.50, 1.00, 1.50, 1.95) in LUT, but the AERONET data were available in 340 nm, 380 nm, 440 nm, 500 nm, 675 nm, 870 nm, 936 nm, 1020 nm, and 1640 nm wavelength channels (no AOD observation value in 550 nm). Only AOD values of the same observation band could be compared, so the AOD values of AERONET between different observation band channels needed to be converted. We used the Angstrom formula to convert [71]:

(4) $$\tau_{\lambda }=\beta *\lambda^{-\alpha }$$

where λ is the wavelength; $\tau_{\lambda }$ is AOD of the wavelength λ; β is the atmospheric turbidity index; and α is the wavelength index.

#### 2.3.2. AOD-PM_2.5_ Spatial-Temporal Regression Models

When observing the relationships between AOD samples and PM_2.5_ samples, we found that PM_2.5_ tended to increase gradually with increasing AOD. Therefore, AOD samples were taken as the independent variable and PM_2.5_ samples as the dependent variable. In order to infer the relationship between AOD (independent variable) and PM_2.5_ (dependent variable), we mainly used 6 linear and nonlinear regression models, including linear, logarithmic, exponential, power, quadratic polynomial, and cubic polynomial function regression models (Table 4). By combining terrain, landscape, and other geospatial information, we applied and tested each model pixel by pixel based on seasonal characteristics and the actual situation in SHB.

The first part of the AOD samples (estimated from the MODIS images) and the PM_2.5_ samples (observed from the monitoring stations) was used for model modeling, and the second part was used for model testing. The optimal model was determined based on model fit (determinant coefficient, R^2^) and error results (root mean square error, RMSE) (Equations (5) and (6)) to build a spatial-temporal estimation model of AOD-PM_2.5_ suitable for SHB and to simulate the spatial-temporal pattern of PM_2.5_ concentration based on the grid:

(5) $$R^{2}=\frac{\sum_{i=1}^{n}{\left({\hat{y}}_{i}-\bar{y}\right)}^{2}}{\sum_{i=1}^{n}{\left(y_{i}-\bar{y}\right)}^{2}}$$

(6) $$RMSE=\sqrt{\frac{\sum_{i=1}^{n}{\left|{\hat{y}}_{i}-y_{i}\right|}^{2}}{n}}$$

where $R^{2}$ is the determine coefficient; RMSE is the root mean square error; n is the sample number; ${\hat{y}}_{i}$ is the value of independent variable i; $y_{i}$ is the value of dependent variable i; and $\bar{y}$ is the mean value of the dependent variable.

#### 2.3.3. Pearson’s and Spearman’s Rank Correlation Coefficients

Pearson’s correlation coefficient (*R*) was applied to the correlation analysis between observation AOD (ground-based from AERONET) and inversion AOD (estimated from MODIS images), seeing by Equation (7):

(7) $$R=\frac{\sum_{i=1}^{n}\left(z_{i}-\bar{z}\right)\left(u_{i}-\bar{u}\right)}{\sqrt{\sum_{i=1}^{n}{\left(z_{i}-\bar{z}\right)}^{2}\sum_{i=1}^{n}{\left(u_{i}-\bar{u}\right)}^{2}}}$$

where R is the Pearson correlation coefficient; n is the sample number; $z_{i}$ is the observation AOD value of i; $\bar{z}$ is the average value of observation AOD; $u_{i}$ is the inversion AOD value of i; and $\bar{u}$ is the average value of inversion AOD.

Spearman’s rank correlation coefficient (ρ) was used to indicate the correlation between estimated AOD and observed PM_2.5_ as in Equation (8):

(8) $$\rho =1-\frac{6\sum_{i=1}^{n}{\left(G_{i}-H_{i}\right)}^{2}}{n\left(n^{2}-1\right)}$$

where ρ is the Spearman’s rank correlation coefficient, $G_{i}\;$ is the rank of estimated AOD; $H_{i}$ is the rank of observed PM_2.5_ value; is the rank difference of estimated AOD and observed PM_2.5_ value.

#### 2.3.4. Relative Exposure Risk Model

Based on the estimated PM_2.5_ concentration and population density data at 1-km grid level, this study used the relative exposure risk model to assess the resident exposure level to PM_2.5_ air pollution [61,72], as in Equation (9):

(9) $$Q_{i}=\frac{P_{i}\times M_{i}}{\sum_{i=1}^{n}P_{i}\times \frac{M_{i}}{n}}$$

where i is the grid number; $Q_{i}$ is the relative population exposure risk of i; $P_{i}$ is the population density of i (unit: person/km^2^); $M_{i}$ is the PM_2.5_ concentration of i (unit: µg/m^3^); and n is the total number of grids.

For the convenience of analysis, values of the exposure risk results were divided into six levels referring to previous studies [73,74]: extremely safe ($Q_{i}$ = 0), safe (0 < $Q_{i}$ ≪ 1), relatively safe (1 < $Q_{i}$ ≪ 2); relatively dangerous (2 < $Q_{i}$ ≪ 3), dangerous (3 < $Q_{i}$ ≪ 5), and extremely dangerous ($Q_{i}$ > 5).

#### 2.3.5. Spatial Autocorrelation Analysis

Moran’s ***I*** is used to represent the spatial autocorrelation, including the global Moran’s ***I*** and the local Moran’s ***I***. The global Moran’s ***I*** is for the spatial autocorrelation of variables in the study area as a whole. When the value of ***I*** approaches 1, the correlation of variables in the spatial distribution is more significant; when the value of ***I*** approaches 0, the correlation is weaker. The local Moran’s ***I*** refers to the correlation degree between the local area and the surrounding area, and its results can be shown in the LISA agglomeration figure. Its $I_{i}$ value is calculated by Equations (10) and (11):

(10) $$I_{i}=\frac{\left(x_{i}-\bar{x}\right)}{S_{i}^{2}}\overset{n}{\underset{i,j=1}{\sum }}w_{i,j}\left(x_{j}-\bar{x}\right)$$

(11) $$S_{i}^{2}=\frac{\sum_{i,j=1}^{n}{\left(x_{i}-\bar{x}\right)}^{2}}{n-1}$$

where n is the number of grids, i≠j, $x_{i}$, and $x_{j}$ are values of variables of i and j, $\bar{x}$ is the average value, and $w_{i,j}$ is the weight matrix for the proximity between i and j. On the significance test, the significance level is α = 0.05.

The Results of $I_{i}$ are divided into five types for the spatial agglomeration characteristics: HH (high-high) is the high-value agglomeration phenomenon, LL (low-low) is the low-value agglomeration phenomenon, LH (low-high) and HL (high-low) are high values alternate with low values, and NS (not significant) is no obvious agglomeration features.

## 3. Results

### 3.1. AOD Inversion Results

The value range of AOD inversion results was from 0 to 1.95, which was a dimensionless value. The larger the AOD was, the greater the aerosol optical thickness was, indicating the lower atmospheric transmittance. The 0 value indicated the existence of no aerosol particles, which was the best atmospheric condition and indicated that solar radiation was not reduced when it passed through the atmosphere.

Because the remote sensing images were large and susceptible to cloud influence, the AOD inversion process and results would have been affected by large areas of cloud in those time periods. We screened MODIS images from all of the year of 2016 one by one, and we excluded images that were seriously blurred by clouds. Finally, we obtained monthly AOD results for SHB (from average daily AOD) for February, March, April, May, June, July, August, September, November, and December (10 months in total) and seasonal AOD results (from average monthly AOD) for spring (March, April, May), summer (June, July, August), autumn (September, November), and winter (February, December) in SHB.

#### 3.1.1. Monthly AOD Results

**Figure 3:**
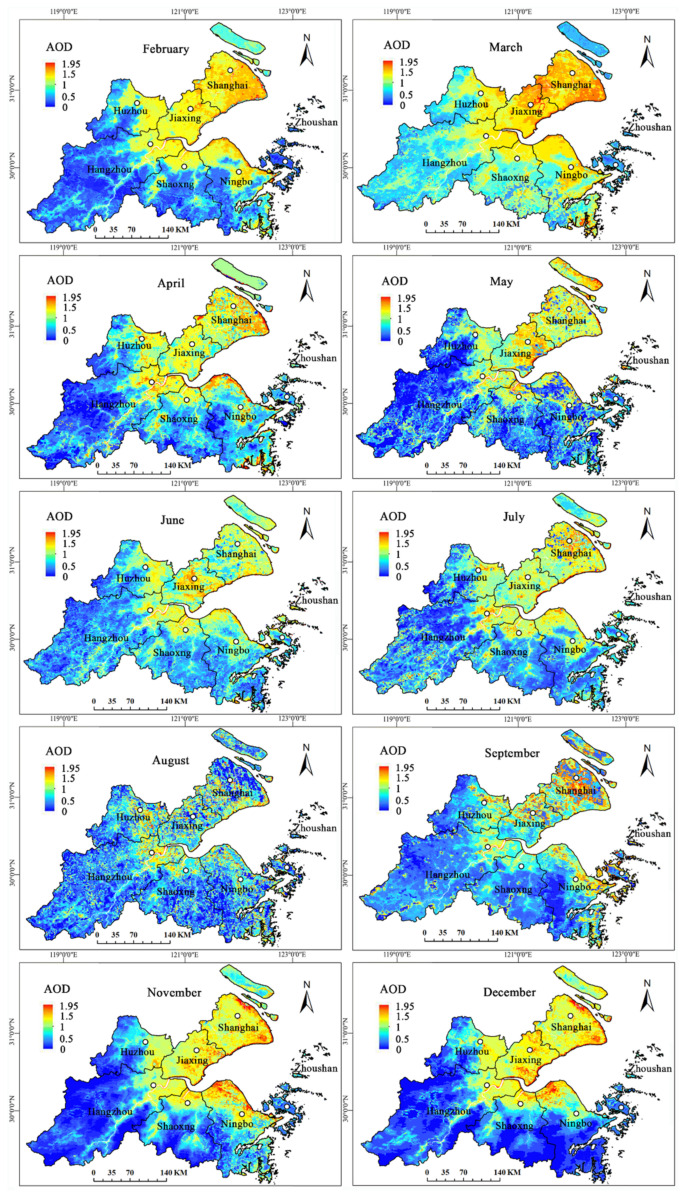
Monthly inversion AODs in SHB.

In the monthly AOD results in Figure 3, higher AOD values were concentrated in Shanghai, Jiaxing, northwest of Hangzhou, Shaoxing, and northern Ningbo, and lower AODs were concentrated in southwest and south of SHB, namely, southwest of Hangzhou, south of Shaoxing and Ningbo, and Zhoushan. Because some MODIS images in a certain time were affected by cloud interference, a small portions of them were eliminated and then showed null values. However, this did not affect the inversion results for other regions, so they continued to participate in the calculation. In 2016, the AOD began increasing steadily beginning in February through May and gradually decreased in June and July, reached the lowest in August; then they gradually increased from September to November and reached the peak in December. Therefore, AOD showed a time variation tendency of “high-low-high” in SHB.

#### 3.1.2. Seasonal AOD Results

**Figure 4:**
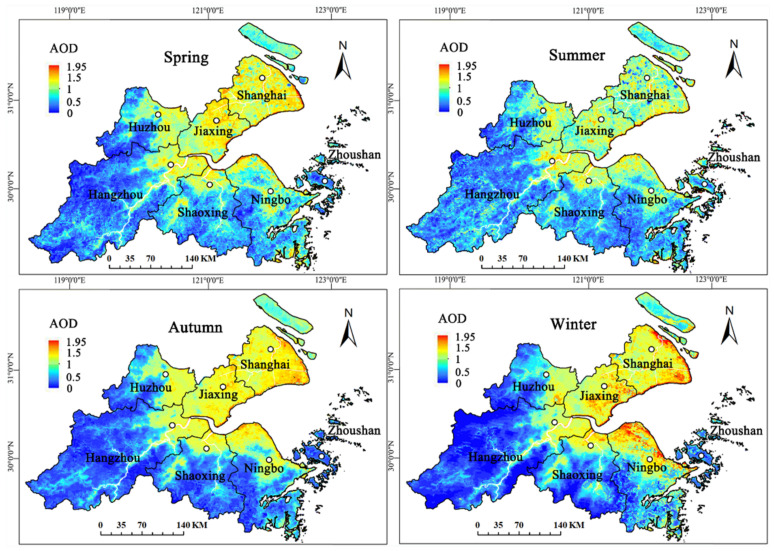
Seasonal inversion AODs in SHB.

In the seasonal AOD results in Figure 4, inversion AOD still presented the distribution characteristics of higher at the coast and lower in the south in spatial scale. In terms of time scale, the overall AOD was lowest in summer and highest in winter, with little difference between spring and autumn, and seasonal AOD still presented a high-low-high tendency in SHB. For higher-value coastal regions, the distribution of low and middle values was interphase in summer, the distribution of middle and high values was interphase in winter, and the distribution of middle values was uniform in spring and autumn, indicating that AODs in the coastal regions were greatly different in summer and winter. Moreover, Zhoushan had the lowest AOD throughout all four seasons, indicating the least PM_2.5_ pollution and the best air quality in 2016.

#### 3.1.3. Verification Result

Using Level 1.5 AERONET AOD data (from ground-based AOD observation stations) of “SONET_Shanghai” and “SONET_Zhoushan”, we verified the accuracy of the inversion AODs. In 2016, there were 29 days of Level 1.5 data available for SHB. AOD inversion values on those days were extracted through software, and then invalid AOD values (disturbed by clouds) were removed to obtain valid values. Finally, values of inversion AOD and observation AOD could be used for verification, a total of 21 groups of data (Table 5).

The verification results in Table 5 show that inversion AOD and observation AOD in terms of time trend were approximately the same. From the Pearson’s correlation analysis, M and SD for the inversion AOD were higher than those for the observation AODs, and R was 0.781 at the 0.01 significance level, showing that the observation AODs were strongly related to the inversion AODs. The AOD result was at a spatial resolution of 1 km × 1 km. Therefore, we verified that the inversion AODs obtained by MODIS data and EDTA were relatively accurate, and the inversion results could be used for the estimation of PM_2.5_ concentrations at higher accuracy and resolution.

### 3.2. Seasonal Spatial-Temporal Models

#### 3.2.1. Correlation Analysis of Inversion AOD and Observation PM_2.5_

**Figure 5:**
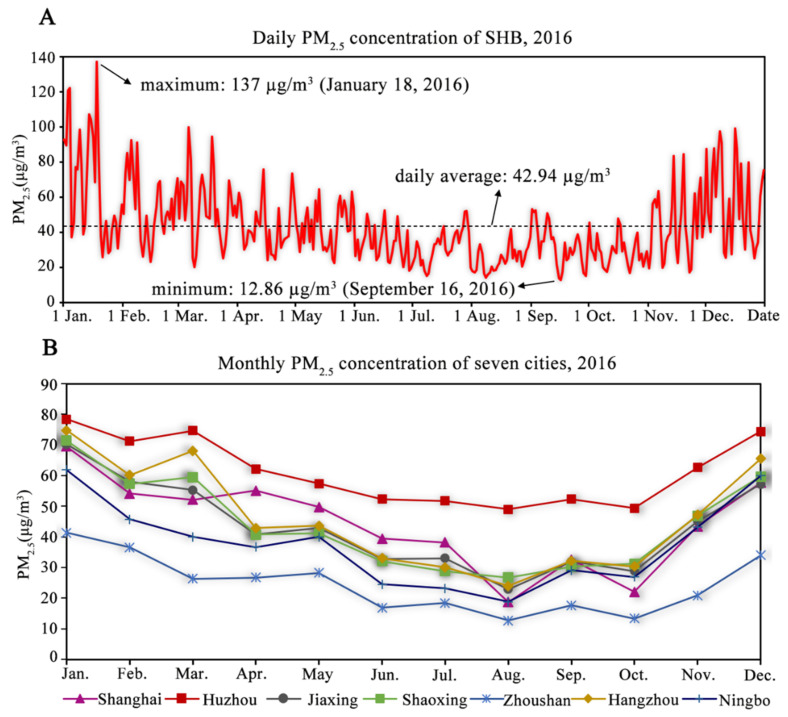
Observations of PM_2.5_ in SHB. (**A**). Daily average PM_2.5_ concentration. (**B**). Monthly average PM_2.5_ concentration of each city.

**Figure 6:**
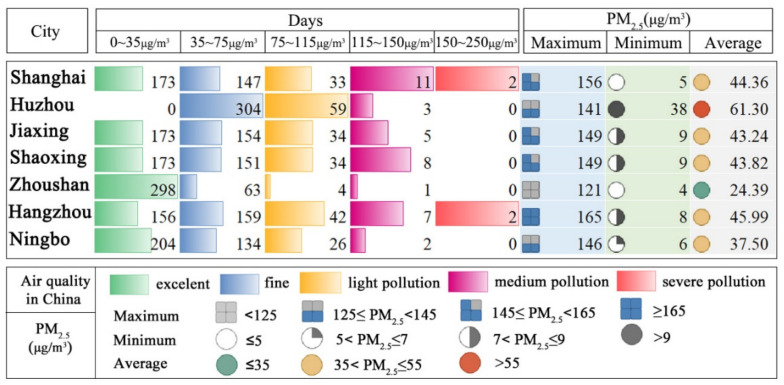
Air quality ratings and observed PM_2.5_ concentrations in seven cities.

Observation PM_2.5_ (from ground-level monitoring stations) was used to explore the relationship with inversion AOD because of its uneven distribution in space. In the observation PM_2.5_ data (Figure 5A), the daily average PM_2.5_ concentration was 42.94 µg/m^3^ in 2016, with the highest being 137 µg/m^3^ in January and the lowest being in 12.86 µg/m^3^ in September. PM_2.5_ concentration decreased gradually from winter to summer and increased gradually from summer to winter. Among all cities (Figure 5B), Huzhou ranked first in 2016, with a monthly average PM_2.5_ concentration of 61.30 µg/m^3^, followed by Hangzhou at 45.99 µg/m^3^, Shanghai at 44.36 µg/m^3^, Shaoxing at 43.82 µg/m^3^, Jiaxing at 43.24 µg/m^3^, and Ningbo at 37.50 µg/m^3^. Concentrations were highest in winter (January and December) and lowest in summer (August). According to the PM_2.5_ concentration air quality standards in China, Zhoushan had the lowest PM_2.5_ concentration of 24.39 µg/m^3^ and the most air quality standard days (361 days, PM_2.5_<75 µg/m^3^)(Figure 6), indicating it was the least polluted city in SHB. Shanghai and Hangzhou had two days of severe pollution (150 < PM_2.5_ ≤ 250 µg/m^3^), with maximum PM_2.5_ concentrations of 156 and 165 µg/m^3^, respectively. The number of days exceeding the standard from most to least occurred in Huzhou (62 days), Hangzhou (51 days), Shanghai (46 days), Shaoxing (42 days), Jiaxing (39 days), Ningbo (28 days), and Zhoushan (5 days) in 2016 (Figure 6).

Values for inversion AOD and observation PM_2.5_ were processed by the min-max normalization method using a total of 410 groups (Table 6). The monthly correlation analysis results showed inversion AOD had the best correlation with observation PM_2.5_ in May, with ρ of 0.631 (at 0.01 confidence level), followed by in August, with ρ of 0.607. Since there was a big difference between inversion AOD and observation PM_2.5_ values every month in 2016, the correlations between them were calculated by season. In the seasonal correlation analysis in Table 6, we found that the best correlation was in summer, with ρ of 0.684 (at 0.01 confidence level), followed by spring, with ρ of 0.538. Overall, PM_2.5_ concentration increased gradually with the increase in AOD. The correlation in each season was better than that in each month. Therefore, seasonal modeling was more effective for PM_2.5_ estimation in SHB.

#### 3.2.2. Seasonal Model Building and Verification

Based on the correlation analysis of inversion AOD and observation PM_2.5_, we obtained 123 groups of data in spring, 123 groups in summer, 82 groups in autumn, and 82 groups in winter. A part of each season’s data was used for model building, and another part was used for model verification. AOD was the independent variable and PM_2.5_ the dependent variable. R^2^ represented the model fit degree, and F is for the significance of the model. The larger these values, the more significant and suitable the model is.

**Figure 7:**
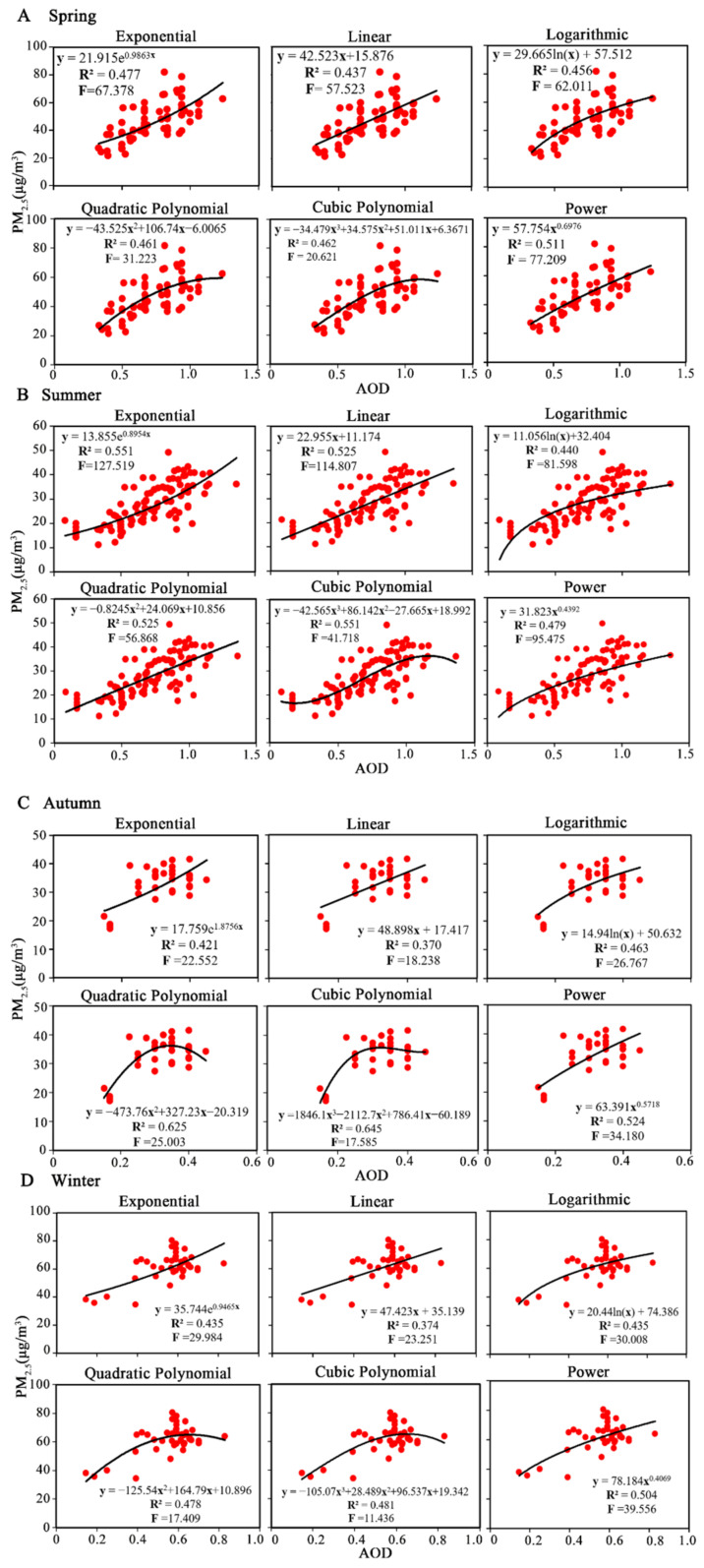
Seasonal model-building results. (**A**–**D**) for spring, summer, autumn, winter, respectively.

In spring, the first 82 groups were used for model building, and the last 35 groups were used for model verification (6 groups of abnormal values eliminated). The seasonal model building results (Figure 7A) show that the power model was the best in spring, with R^2^ = 0.511 and F = 77.209. In summer, the first 84 groups were used for model building, and the last 35 groups were used for model verification (4 groups of abnormal values eliminated), and the exponential model was the best in summer, with R^2^ = 0.551 and F = 127.519 (Figure 7B). In autumn, the first 41 groups were used for model building, and the last 41 groups were used for model verification, and the power model was the best, with R^2^ = 0.524 and F = 34.180 (Figure 7C). In winter, the first 41 groups were used for model building, and the last 41 groups were used for model verification, and the power model was the best, with R^2^ = 0.504 and F = 39.556 (Figure 7D).

The seasonal model verification results in Table 7 show that the power model had better fit and the fewest errors in spring, with R^2^ = 0.513 and RMSE = 6.204; the exponential model showed the best fit and the smallest error in summer, with R^2^ = 0.640 and RMSE = 3.979; the fit of the power model was the best and the error was smaller in autumn, with R^2^ = 0.520 and RMSE = 7.893; and the power model had better fit and minimum error in winter, with R^2^ = 0.540 and RMSE = 7.392. Overall, the power model was the best in spring, autumn, and winter and the exponential model was the most suitable for summer, showing that both played significant roles in estimating PM_2.5_ concentration, especially the power model. Based on these optimal seasonal models, we combined observation PM_2.5_ concentrations and the geographic big data according to the actual situation and features in SHB to produce seasonal spatial-temporal models for PM_2.5_ estimation.

### 3.3. PM_2.5_ Estimation Results

**Figure 8:**
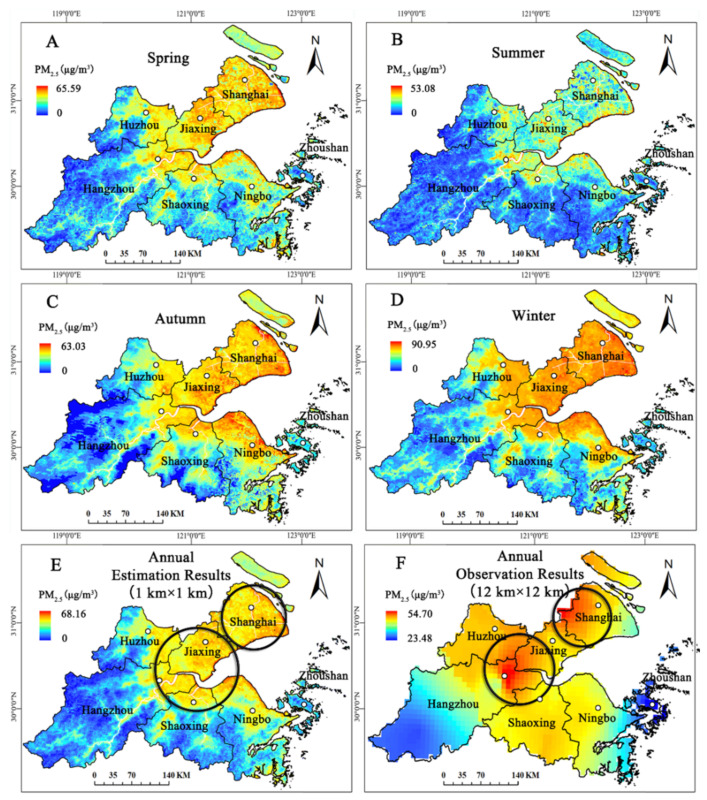
2016 PM_2.5_ concentration maps for SHB. (**A**–**D**) Seasonal estimates of PM_2.5_ concentration. (**E**) Annual average estimates of PM_2.5_ concentration (1 km × 1 km). (**F**) Observation PM_2.5_ concentration interpolation results (12 km × 12 km).

Based on these seasonal spatial-temporal models, we obtained seasonal PM_2.5_ estimation results for SHB (Figure 8). Spatially, the PM_2.5_ concentrations in each season showed the same distribution characteristic of higher in the coastal areas and lower in the mountainous areas. Zhoushan was the city with the lowest PM_2.5_ concentration in all seasons, which was always lower than 40 µg/m^3^. Shanghai and Jiaxing were both above 40 µg/m^3^ in all seasons, making them the two most polluted cities. From the perspective of time, the average PM_2.5_ concentrations over the four seasons was winter > spring > autumn > summer, and the PM_2.5_ concentration showed a high-low-high tendency in a year.

From the annual average PM_2.5_ results in Figure 8E, we see that the PM_2.5_ concentration range was 0~68.16 µg/m^3^, and its spatial resolution was 1 km × 1 km. Concentrations were high (50~70 µg/m^3^) in Shanghai, Jiaxing, northwest of Hangzhou, Shaoxing, and the northern part of Ningbo; medium (30~50 µg/m^3^) in the vicinity of high concentration; and low (0~30 µg/m^3^) mainly in the southwest of Hangzhou and Zhoushan. Overall, the spatial distribution of PM_2.5_ concentrations was higher in the northeast and lower in the southwest, indicating that the area along the Hangzhou Bay was seriously affected by fine particles, and the air quality in the mountainous area with higher altitude was better. This correlated with the elevation characteristics of SHB (Figure 1). The coastal areas are of low altitude, with dense populations, convenient transportation, and developed industry and commerce, which is not conducive to haze diffusion. However, the mountainous areas are of high altitude, with high vegetation coverage and abundant rain, so there is less PM_2.5_ pollution and good air quality.

In this work, the spatial distribution result of observation PM_2.5_ concentration was obtained by the Kriging interpolation of GIS, and its optimal spatial scale could only reach 12 km × 12 km (because of the small number of monitoring stations) (Figure 8F). Compared with estimates of PM_2.5_ concentrations from remote sensing data (1 km × 1 km), although PM_2.5_ concentrations are high in similar areas (Shanghai, the junctional zone of Jiaxing, Huzhou, and Hangzhou), the estimated PM_2.5_ concentrations are more continuous and even more accurate in some areas, such as east and west of Huzhou and south and north of Shaoxing. Eastern Huzhou has a larger population and a more developed economy, so the impacts of PM_2.5_ population on eastern areas should be greater than that on western areas. The same is true in the northern and southern areas of Shaoxing. Therefore, PM_2.5_ concentration estimates from remote sensing images are closer to the real situation in SHB, reflecting the necessity of remote sensing to estimate PM_2.5_ for assessing fine pollution levels at a regional scale.

### 3.4. Exposure Risk Assessment

**Figure 9:**
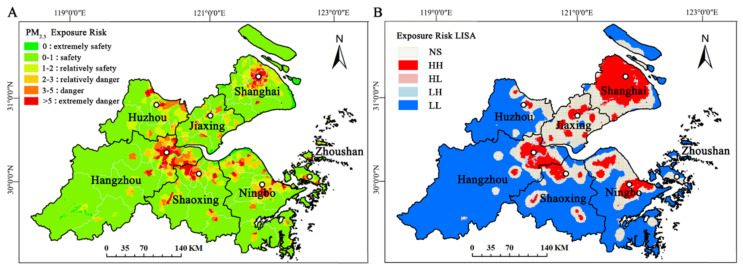
Maps of PM_2.5_ exposure risk (**A**) and LISA agglomeration (**B**) in SHB, China.

Based on population density data from Worldpop (1-km grid level) and the relative exposure risk model, the exposure risk to PM_2.5_ pollution was evaluated using GIS. Figure 9A shows that each level of average annual exposure risk was unevenly distributed around the cities, indicating that PM_2.5_ air pollution degree differs greatly around SHB. The range of exposure risk values was from 0 to 133, with a large difference between the lowest value and the highest value. In addition, about 2/3 of the land area was a safe zone, and the rest was in a danger zone (all low-elevation urban areas). By city, the average annual exposure risk was Shanghai (3.57) > Jiaxing (1.24) > Ningbo (0.77) > Shaoxing (0.51) > Hangzhou (0.50) > Zhoushan (0.46) > Huzhou (0.44). Except for Shanghai, other cities were in the safe range, with residents of Huzhou city the least exposed to PM_2.5_ pollution. Moreover, the danger level of exposure risk in each city was concentrated in the coastal areas around SHB, and the higher elevations (in the south) were all safe, with the lowest PM_2.5_ pollution degree. The highest danger level was mainly concentrated in the main urban areas, with Shanghai and Hangzhou as the most serious.

On the global Moran’s ***I*** for SHB, the values were 0.6436 in spring, 0.6174 in summer, 0.6365 in autumn, and 0.6351 in winter. Values of *p* for all four seasons were all above 0.01, indicating strong positive spatial correlations and high significance of PM_2.5_ exposure risk in SHB. The results of the LISA spatial aggregation (Figure 9B) indicated that the annual average PM_2.5_ exposure risk in SHB had a strong spatial autocorrelation and an obvious spatial aggregation. The high-value agglomeration (HH) was distributed in Shanghai, northeastern Hangzhou, central Jiaxing, and central Ningbo. The low-value agglomeration (LL) was mainly distributed in the areas with higher elevations, Shanghai and Zhoushan City in the Chongming District. Overall, the coastal areas were dominated by high-value clustering, and mountainous areas were dominated by low-value clustering.

## 4. Discussion

Due to the high cost of construction and maintenance of air quality monitoring stations, the number of stations is small in China. Monitoring stations are unevenly distributed, mostly concentrated in relatively developed areas. The observation PM_2.5_ concentrations could only reflect the local PM_2.5_ situations but could not truly reflect the actual characteristics of spatial distribution. In addition, there are few available, suitable, and high spatial resolution PM_2.5_ products, so we use daily MODIS images and introduce the EDTA method to retrieve high-resolution AODs in space and time to create high-precision and high-resolution PM_2.5_ concentrations at a 1-km grid level based on a seasonal spatial-temporal estimation model. In this work, we addressed problems of the spatial resolution and the spatial-temporal continuity of PM_2.5_ concentration data.

Previous studies that adopt spatial distribution analysis of PM_2.5_ using monitoring station data and GIS interpolation methods only account for local area situations or analyze variations in PM_2.5_ characteristics and time trends using annual average data. Studies using monitoring stations to obtain PM_2.5_ did not take suitable spatial resolution into account; studies using interpolation methods to derive PM_2.5_ did not focus on seasonal differences in PM_2.5_ pollution. Both are not precise enough in time and space.

By introducing the EDTA method and seasonal spatial-temporal models, we estimate the seasonal PM_2.5_ concentration in a high spatial resolution of 1 km × 1 km. The verification accuracy (R^2^) of estimation PM_2.5_ concentration reached 0.513 in spring, 0.640 in summer, 0.520 in autumn, and 0.540 in winter, and the estimation error (RMSE) was in the range of 3.979~7.893 μg/m^3^. We showed the feasibility and reliability of retrieving AOD and estimating PM_2.5_ from MODIS remote sensing images. According to the characteristics of different seasons, we also constructed a corresponding estimation model that has seasonal applicability. Today, 1 km-grid level of PM_2.5_ concentration is a higher-resolution data source, which could allow for assessing fine PM_2.5_ pollution at small and medium scale. These data sets and research results are useful for policymakers in air pollution control administration to plan a more sustainable living environment.

SHB is seriously affected by the southeast monsoon and plum rain season in summer. Despite the high time resolution of the MODIS images (daily), these images are often influenced by massive cloud cover in AOD inversion and PM_2.5_ estimation. leading to poor estimated results on some days. Therefore, the replacement or supplement of these data (seriously disturbed by clouds) will be helpful for improving the accuracy of AOD inversion and PM_2.5_ estimation. While the inversion AOD accuracy is high by the method of EDTA, the basic DTA method has certain requirements for the vegetation coverage in the region. In high surface reflectance areas, this method is only moderately effective. Combining more suitable inversion methods according to different surface types could increase the accuracy.

Future investigations would be helpful in the following aspects: changes in particulate matter at a micro scale would improve the accuracy of AOD-PM_2.5_ estimation models; problems of data loss and signal-noise ratio (caused by cloud and rainfall interference) could be anticipated in the development of multi-source spatial-temporal data fusion; for instance, we are trying to solve the single-source data in the product resolution and spatial-temporal coverage and improve the accuracy and reliability of estimated PM_2.5_ data; improved estimation PM_2.5_ data could be used to evaluate the population exposure risk to PM_2.5_, health economic losses, and early warning and prevention, in order to provide scientific reference for policymakers for improving urban atmospheric pollution and living environments.

## 5. Conclusions

This study developed a framework to improve the spatial resolution of AOD and PM_2.5_ dataset and present the health risk assessment from PM_2.5_ pollution: first, we retrieve the daily AOD using MODIS remote sensing images, AERONET AOD data, and the Enhanced Dark Target Algorithm (EDTA). Then we mapped the monthly and seasonal AOD results at a spatial resolution of 1 km × 1 km. Second, we inferred the optimal relationship between retrieved AOD and observed PM_2.5_ in four seasons, and spatial-temporal seasonal models were developed to estimate PM_2.5_ concentration. According to geographical features and seasonal characteristics in the study area, we obtain seasonal PM_2.5_ at 1-km grid level by GIS platform. Third, we assessed health risk from PM_2.5_ pollution using high-accuracy population density data and the relative exposure risk model. Last, the usage and deficiency of PM_2.5_ dataset and risk assessment results were discussed. Therefore, reasonable assessments on health risk from PM_2.5_ pollution are important for improving public health and living environment.

## Figures and Tables

**Table 1:** Data from AERONET AOD stations in SHB in 2016.

City	Site	Longitude (°E)	Latitude (°N)	Data
Shanghai	SONET_Shanghai	121.481	31.284	Level 1.0 ^a^, Level 1.5 ^b^
	Shanghai_Minhang	121.397	31.130	null
	Shanghai_Met	121.549	31.221	null
Hangzhou	LA-TM	119.440	30.324	null
	Hangzhou-ZFU	119.727	30.257	null
	Hangzhou_City	120.157	30.290	null
	Qiandaohu	119.053	29.556	null
Ningbo	Ningbo	121.547	29.860	null
Zhoushan	SONET_Zhoushan	122.188	29.994	Level 1.0 ^a^, Level 1.5 ^b^

^a^ Level 1.0 for original data. ^b^ Level 1.5 for cloud filtering and quality control data.

**Table 2:** Geographical coordinates of PM_2.5_ monitoring stations in SHB.

City	Monitoring Station	Longitude (°E)	Latitude (°N)
Shanghai	Putuo	121.3984	31.2637
	NO.15 Factory	121.3614	31.2228
	Hongkou	121.4919	31.2825
	Shanghai Normal University	121.4232	31.1675
	Sipiao	121.5360	31.2659
	Dianshan Lake	120.9382	31.0927
	Jingan	121.4363	31.2305
	Chuansha	121.7042	31.1994
	Pudong New Area	121.6634	31.2428
	Zhangjiang	121.5918	31.2108
Jiaxing	Qinghe Primary School	120.7543	30.7819
	Jiaxing College	120.7372	30.7517
	Disabled Persons’ Federation	120.7739	30.7601
Hangzhou	Binjiang	120.1924	30.1876
	Xixi	120.1000	30.2645
	Qiandao Lake	119.0214	29.6020
	Xiasha	120.3442	30.3221
	Wolong Bridge	120.1385	30.2493
	Zhejiang Agricultural University	119.7355	30.2621
	Zhaohui NO.5 Community	120.1688	30.2940
	Hemu Primary School	120.1312	30.3161
	Linping	120.3133	30.4272
	Chengxiang	120.3052	30.2615
	Yunqi	120.1010	30.1989
Shaoxing	Paojiang	120.6238	30.0842
	East Management Committee of Development Zone	120.8460	29.5986
	Shuxia Wang	120.5828	30.0159
Ningbo	Environmental Protection Building	121.5865	29.8582
	Wanli College	121.5695	29.8230
	Longsai Hospital	121.7223	29.9596
	Sanjiang Middle School	121.5647	29.8940
	Qiangtang Waterwork	121.6440	29.7770
	Taigu Primary School	121.5985	29.8596
	Environmental Monitoring Center	121.5351	29.8709
	Wanli International School	121.6234	29.9019
Zhoushan	Dinghai TanFeng	122.1320	30.0240
	Putuo Donggang	122.3285	29.9791
	Lincheng New Area	122.2020	29.9885
Huzhou	Renhuangshan New Area	120.0976	30.9000
	West Waterwork	120.0844	30.8811
	Wuxing	120.1158	30.8710

**Table 3:** Geometric parameters of LUT based on a 6 S transmission model.

Major Parameters	Settings
Satellite zenith angle	0°, 12°, 24°, 36°, 48°, 60°
Solar zenith angle	0°, 12°, 24°, 36°, 48°, 60°
Relative azimuth angle	0~180°, 24° (interval)
AOD at 550 nm wavelength	0, 0.25, 0.50, 1.00, 1.50, 1.95
Central wavelength	470 nm, 660 nm, 2100 nm
Elevation	0
Surface type	Vegetation

**Table 4:** Regression models for AOD-PM_2.5_ relationship prediction.

Regression Model	Equation
Linear	y = a_0_ + a_1_x
Logarithmic	y = a_0_ + a_1_ln(x)
Exponential	y = a_0_ × e^a1x^
Power	y = a_0_(x^a1^)
Quadratic Polynomial	y = a_0_ + a_1_x + a_2_x^2^
Cubic Polynomial	y = a_0_ + a_1_x + a_2_x^2^ + a_3_x^3^

x for independent variable. y for dependent variable. a_0_, a_1_, a_2_, a_3_ for relevant parameters.

**Table 5:** Pearson correlation analysis of inversion AOD and observation AOD.

Site	Days	Date	AOD Value	
			Inversion	Observation
SONET_Shanghai	10	1 May 2016	0.610	0.785
		3 May 2016	0.792	0.890
		4 May 2016	0.500	0.449
		12 May 2016	0.375	0.304
		15 May 2016	0.400	0.551
		16 May 2016	0.917	0.346
		17 May 2016	0.400	0.222
		24 May 2016	1.170	1.194
		25 May 2016	1.246	0.951
		6 June 2016	0.720	1.153
SONET_Zhoushan	11	30 April 2016	0.808	0.464
		1 May 2016	0.730	0.474
		3 May 2016	0.320	0.314
		4 May 2016	0.700	0.775
		11 May 2016	1.170	0.815
		12 May 2016	0.563	0.534
		16 May 2016	0.200	0.218
		17 May 2016	0.150	0.154
		18 May 2016	0.200	0.199
		24 May 2016	1.000	1.022
		6 June 2016	0.350	0.360
M ^a^			0.634	0.580
SD ^b^			0.334	0.328
R ^c^			0.781	0.781
Significant (bilateral)			0	0

^a^ M for mean value. ^b^ SD for standard deviation. ^c^ R for Pearson correlation coefficient.

**Table 6:** Correlation analysis of inversion AOD and observation PM_2.5_ (at 0.01 confidence level).

Month	Sample	$\rho {\;}^{a}$	N ^b^	Season	Sample	$\rho {\;}^{a}$	N ^b^
March	AOD	0.021	41	Spring		0.538	123
	PM_2.5_						
April	AOD	0.406	41		AOD		
	PM_2.5_				PM_2.5_		
May	AOD	0.631	41				
	PM_2.5_						
June	AOD	0.443	41	Summer		0.684	123
	PM_2.5_						
July	AOD	0.432	41		AOD		
	PM_2.5_				PM_2.5_		
August	AOD	0.607	41				
	PM_2.5_						
September	AOD	0.395	41	Autumn		0.474	82
	PM_2.5_				AOD		
November	AOD	0.138	41		PM_2.5_		
	PM_2.5_						
December	AOD	0.314	41	Winter		0.341	82
	PM_2.5_				AOD		
February	AOD	0.121	41		PM_2.5_		
	PM_2.5_						

^a^ ρ for Spearman’s rank correlation coefficient. ^b^ N for sample size.

**Table 7:** Seasonal model building and verification results.

Season	Model	Equation	Model Building		Model Verification	
			R^2^	F	R^2^	RMSE
Spring	A ^a^	y = 42.523x + 15.876	0.437	57.523	0.514	6.587
	B ^b^	y = 29.665ln(x) + 57.512	0.456	62.011	0.503	6.719
	C ^c^	y = 21.915e^0.9863x^	0.477	67.378	0.504	6.246
	D ^d^	y = −43.525x^2^ + 106.74x − 6.0065	0.461	31.223	0.506	6.829
	E ^e^	y = −34.479x^3^ + 34.575x^2^ + 51.011x + 6.3671	0.462	20.621	0.515	6.900
	F ^f^	y = 57.754x^0.6976^	0.511	77.209	0.513	6.204
Summer	A ^a^	y = 22.955x + 11.174	0.525	114.807	0.590	4.432
	B ^b^	y = 11.056ln(x) + 32.404	0.440	81.598	0.418	5.254
	C ^c^	y = 13.855e^0.8954x^	0.551	127.519	0.640	3.979
	D ^d^	y = −0.8245x^2^ + 24.069x + 10.856	0.525	56.868	0.588	4.440
	E ^e^	y = −42.565x^3^ + 86.142x^2^ − 27.665x + 18.992	0.551	41.718	0.606	4.113
	F ^f^	y = 31.823x^0.4392^	0.479	95.457	0.518	4.313
Autumn	A ^a^	y = 48.898x + 17.417	0.370	18.238	0.488	8.857
	B ^b^	y = 14.94ln(x) + 50.632	0.463	26.767	0.515	7.534
	C ^c^	y = 17.759e^1.8756x^	0.421	22.552	0.478	8.980
	D ^d^	y = −473.76x^2^ + 327.23x − 20.319	0.625	25.003	0.455	9.010
	E ^e^	y = 1846.1x^3^ − 2112.7x^2^ + 786.41x − 60.189	0.645	17.585	0.497	9.087
	F ^f^	y = 63.391x^0.5718^	0.524	34.180	0.520	7.893
Winter	A ^a^	y = 47.423x + 35.139	0.373	23.251	0.508	7.957
	B ^b^	y = 20.44ln(x) + 74.386	0.435	30.008	0.547	7.621
	C ^c^	y = 35.744e^0.9465x^	0.435	29.984	0.471	7.706
	D ^d^	y = −125.54x^2^ + 164.79x + 10.896	0.478	17.409	0.550	7.450
	E ^e^	y = −105.07x^3^ + 28.489x^2^ + 96.537x + 19.342	0.481	11.436	0.553	7.429
	F ^f^	y = 78.184x^0.4069^	0.504	39.556	0.540	7.392

x: independent variable. Y: dependent variable. ^a^ A: The linear regression model. ^b^ B: The logarithmic regression model. ^c^ C: The exponential regression model. ^d^ D: The quadratic polynomial regression model. ^e^ E: The cubic polynomial regression model. ^f^ F: The power regression model.

## Data Availability

Data is available on request to the corresponding author.

## Abbreviations

GIS	Geographic Information Systems
RS	Remote sensing
SDGs	Sustainable Development Goals
PM_2.5_	fine particulate matter (a diameter of less than 2.5 μm)
GOES	Geostationary Operational Environmental Satellite
METOP	European new generation weather operational satellites
PARASOL	Polarization and Anisotropy of Reflectances for Atmospheric Sciences coupled with Observations from a Lidar
MODIS	Moderate-resolution Imaging Spectror
AVHRR	Advanced Very High Resolution Radiometer
SeaWiFS	Sea-viewing Wide Field of View Sensor
POLDER	Polarization and Directionality of the Earth’s Reflectances
AOD	Aerosol Optical Depth
GEOS	Geosynchronous Earth Orbit Satellite
RAMS	Regional Atmospheric Modeling System
GLM	Generalized Linear Model
GAM	Generalized Additive Models
GWR	Geographically Weighted Regression
ML	Machine Learning
DTA	Dark Target Algorithm
EDTA	Enhanced Dark Target Algorithm
SHB	Shanghai-Hangzhou Bay
NSMS	National Standard Map Service platform in China
ESDC	Environmental Sciences and Data Center in China
NASA	National Aeronautics and Space Administration
LAADS	the Level-1 and Atmosphere Archive and Distribution System
AERONET	Aerosol Robotic Network
CEME	China Environmental Monitoring Center
LUT	Lookup Table
GCP	Ground Control Points
HDF	Hierarchical Data File
